# Microfluidic System Based on Flexible Structures for Point-of-Care Device Diagnostics with Electrochemical Detection

**DOI:** 10.3390/bios15080483

**Published:** 2025-07-24

**Authors:** Kasper Marchlewicz, Robert Ziółkowski, Kamil Żukowski, Jakub Krzemiński, Elżbieta Malinowska

**Affiliations:** 1 Chair of Medical Biotechnology, Faculty of Chemistry, Warsaw University of Technology, Stanisława Noakowskiego 3, 00-664 Warsaw, Poland; elzbieta.malinowska@pw.edu.pl; 2 Centre for Advanced Materials and Technologies (CEZAMAT), Warsaw University of Technology, Poleczki 19, 02-822 Warsaw, Poland; k.zukowski@cezamat.eu (K.Ż.); jakub.krzeminski@pw.edu.pl (J.K.)

**Keywords:** biosensor, electrochemical sensor, *Corynebacterium diphtheriae*, microfluidic device

## Abstract

Infectious diseases poses a growing public health challenge. The COVID-19 pandemic has further emphasized the urgent need for rapid, accessible diagnostics. This study presents the development of an integrated, flexible point-of-care (POC) diagnostic system for the rapid detection of *Corynebacterium diphtheriae*, the pathogen responsible for diphtheria. The system comprises a microfluidic polymerase chain reaction (micro-PCR) device and an electrochemical DNA biosensor, both fabricated on flexible substrates. The micro-PCR platform offers rapid DNA amplification overcoming the time limitations of conventional thermocyclers. The biosensor utilizes specific molecular recognition and an electrochemical transducer to detect the amplified DNA fragment, providing a clear and direct indication of the pathogen’s presence. The combined system demonstrates the effective amplification and detection of a gene fragment from a toxic strain of C. diphtheriae, chosen due to its increasing incidence. The design leverages lab-on-a-chip (LOC) and microfluidic technologies to minimize reagent use, reduce cost, and support portability. Key challenges in microsystem design—such as flow control, material selection, and reagent compatibility—were addressed through optimized fabrication techniques and system integration. This work highlights the feasibility of using flexible, integrated microfluidic and biosensor platforms for the rapid, on-site detection of infectious agents. The modular and scalable nature of the system suggests potential for adaptation to a wide range of pathogens, supporting broader applications in global health diagnostics. The approach provides a promising foundation for next-generation POC diagnostic tools.

## 1. Introduction

The spread of infectious diseases continues to be a major public health risk in the modern world. The COVID-19 pandemic, caused by the SARS-CoV-2 virus, has highlighted the critical importance of rapid diagnostics in protecting public health and preventing the spread of infections. Predicting the emergence of new pathogens and developing strategies to combat them is challenging; however, they are not the only threats. Certain infectious diseases that were previously controlled by global vaccination programs are re-emerging. One such disease is diphtheria, currently diagnosed in several thousand individuals annually [1]. Rapid diagnosis is essential for initiating treatment and controlling the spread of diphtheria.

Traditional diagnostic methods, despite their effectiveness, have limitations such as long processing times and the need for advanced laboratories and well trained personnel. This is particularly problematic in underdeveloped regions with limited access to healthcare facilities [2]. Point-of-care (POC) devices offer an alternative to traditional laboratory diagnostics. These devices enable quick testing at the patient’s location without the need to transport samples. Typically, POC devices are user-friendly and automate the testing procedure. The development of POC platforms is closely linked with lab-on-a-chip (LOC) technology, which allows for the performance of multiple laboratory processes within a small system. LOC devices operate with small volumes of solutions, reducing the costs and time of the diagnostic process. One of the LOC devices is a micro-PCR system used for the detection of pathogens responsible for various infectious diseases [3,4].

Micro-PCR systems offer an alternative to traditional thermocyclers by enabling DNA amplification in a compact device. There are various types of these devices, differing in their approach to reaction processes, heat exchange, flow generation, and materials used for fabrication [5,6,7,8,9]. Most micro-PCR systems have the advantage over traditional thermocyclers, which is a short amplification time of the target DNA fragment. Traditional thermocyclers are limited by the slow temperature changes in heating chambers. Micro-PCR systems overcome this issue by conducting reactions by continuous-flow or using advanced thermal energy exchange methods.

Microfluidic systems are increasingly used in various scientific fields, and modern manufacturing technologies make them easier for large-scale production and commercial use. Despite their advantages, the development of such systems presents numerous challenges such as limited mixing or the unwanted adsorption of reagents. These and other effects must be considered during the design and testing of microsystems, particularly in material selection, fabrication techniques, and microstructure geometry. A significant part of most microsystems, especially those with forced flow, includes components such as pumps and valves. Laboratory scale equipment, often significantly larger than the microsystem itself, is sometimes used for the proper functioning of certain microfluidic systems. However, the miniaturization of flow control elements allows for their integration with microsystems, enabling automation, cost reduction, and application in portable analytical devices, such as POC devices [10].

The detection of DNA that was amplified in PCR reactions can be achieved using DNA biosensors. These sensors operate on the basis of specific molecular interactions between the receptor layer and the analyte for selective detection. Electrochemical transducers are often used in such devices due to their low cost and potential for miniaturization. These features of electrochemical transducers are exceptionally useful for creating portable, disposable systems for pathogen detection at the patient’s location [11]. Designing DNA biosensors for real sample analysis requires careful receptor layer design and optimization of measurement conditions. Key parameters include the sensor’s selectivity for the target pathogen, sensitivity, and detection limit. Ultimately, the most crucial information a biosensor should provide is a simple indication of the presence of the target pathogen DNA in the sample.

In this paper, we present a developed PCR microfluidic device that was fabricated using flexible substrates and was used to perform PCR. The detection of the amplified DNA fragment was performed using the developed biosensor, which was also fabricated using a flexible substrate. This paper shows the ability of the developed system based on flexible structures (PCR microfluidic device integrated with a biosensor) of the rapid and unambiguous amplification and detection of the gene fragment from a toxic strain of *Corynebacterium diphtheriae*, responsible for causing diphtheria. This pathogen was chosen not only due to the increasing number of reported cases but also as a model for developing similar solutions for other infectious diseases. The findings presented in this paper could serve as a foundation for developing other diagnostic systems or devices, as many of the materials, manufacturing techniques, and mechanisms used exhibit a high degree of versatility.

## 2. Materials and Methods

### 2.1. Equipment

The voltammetric measurements were performed using electrochemical workstations CHI 660A and CHI 1040A from CH Instruments. The following measurement techniques were used: Cyclic voltammetry (CV) was conducted at a scan rate of 0.1 V/s. Square wave voltammetry (SWV) was carried out with a pulse amplitude of 25 mV, an increment of 4 mV, and frequencies ranging from 15 Hz to 300 Hz.

Voltammetric measurements were conducted using a three-electrode measurement system consisting of a gold disk working electrode (GDE) from CH Instruments, an Ag/AgCl reference electrode with 1.0 mol/L KCl from Mineral, and a gold wire auxiliary electrode from Sigma Aldrich. Most importantly, three-electrode transducers screen-printed on a flexible substrate were used.

Polymerase chain reaction (PCR) reactions were carried out using the Mastercycler AG 22331 thermal cycler from Eppendorf. The separation of PCR reaction products was achieved using a horizontal gel electrophoresis tank from BIO-RAD. Post-electrophoresis visualization was performed with a UV transilluminator from Hoefer.

A Universal Model VLS 2.30 laser plotter with an additional high-resolution attachment was used for precision cutting and engraving. A CNC micro-milling machine, Datron Model NEO+, was used for fabricating microfluidic device components.

### 2.2. Reagents

The following reagents were used: Hydrophilic polyester film 9984 and double-sided adhesive polyester film 9965, both from 3M and Poly(methyl methacrylate) (PMMA) sheets with thicknesses ranging from 4 to 7 mm (Evonik GS, Essen, Deutschland) were used for PCR microdevice fabrication. Viton™ fluororubber (GG Trading, Jumeirah Lake Towers, Dubai) was used for the fabrication of a seal used in a microvalve. 6-Mercapto-1-hexanol (MCH) (97%) from Sigma Aldrich was used for surface modification of the gold electrode to form a self-assembled monolayer (SAM) that facilitates the immobilization of the receptor DNA probe. Sodium diethyldithiocarbamate trihydrate (DEDTC)·3 H_2_O (>95%) from Sigma Aldrich (St. Louis, USA) was employed for the same purpose. 2-Amino-2-hydroxymethyl-1,3-propanediol (Tris) from Sigma Aldrich was used to prepare buffer solutions, maintaining pH stability during reactions. Ethylenediaminetetraacetic acid (EDTA) (≥98.5%) from Sigma Aldrich was used for electrophoretic procedure. Hydrochloric acid (HCl) (36.5–38.0%) from Sigma Aldrich was utilized for pH adjustment in buffer preparation. Sulfuric acid (H_2_SO_4_) (95.0–98.0%) from Sigma Aldrich was used for the cleaning procedures of the electrode surfaces. Dimethyl sulfoxide (DMSO) (≥99.5%) from Sigma Aldrich was employed as a solvent for various reagents and in the preparation of reaction mixtures. Sodium hydroxide (NaOH) (≥97%) from Sigma Aldrich was used for pH adjustment in buffer solutions. Sodium chloride (NaCl) (≥99%) from Sigma Aldrich was utilized in the preparation of saline solutions and buffers. Potassium chloride (KCl) (≥99%) from Sigma Aldrich was used in buffer solutions. Potassium ferricyanide (K_3_Fe(CN)_6_) (≥99%) from Sigma Aldrich was employed as an electrochemical mediator in redox reactions. Potassium ferrocyanide (K_4_Fe(CN)_6_) (≥98.5%) from Sigma Aldrich was used alongside potassium ferricyanide in electrochemical measurements. Potassium dihydrogen phosphate (KH_2_PO_4_) (≥99%) from Sigma Aldrich was utilized in the preparation of phosphate buffer solutions. Boric acid from Merck (cat. no. STBH1850) was used in buffer preparation and pH control. Hydrogen peroxide (H_2_O_2_) (30%) from POCh was used for cleaning the electrode surfaces. Magnesium chloride (MgCl_2_)·6 H_2_O (≥98%) from Fluka Analytical was added to PCR reactions to stabilize the DNA polymerase activity. Alumina powder with grain sizes of 1 μm, 0.3 μm, and 0.05 μm from Buehler was used for polishing the gold electrode surfaces to ensure a smooth and clean surface for subsequent modifications.

### 2.3. Solutions

The immobilization solution consisted of 1 M potassium dihydrogen phosphate (KH_2_PO_4_) with a pH of 4.5. This solution was used to immobilize the receptor DNA probe on the gold electrode surface.

The hybridization solution contained 10 mM Tris-HCl, 50 mM potassium chloride (KCl), and 2 mM magnesium chloride (MgCl_2_) with a pH of 7.0. This solution facilitated the hybridization process between the target DNA and the immobilized probe.

The piranha solution, composed of hydrogen peroxide (H_2_O_2_) and sulfuric acid (H_2_SO_4_) in a 3:1 ratio, was used for cleaning the gold electrodes, ensuring the removal of organic contaminants and providing a clean surface for subsequent modifications.

The electrochemical measurements utilized a solution of potassium ferricyanide (K_3_Fe(CN)_6_) and potassium ferrocyanide (K_4_Fe(CN)_6_) in 20 mM phosphate-buffered saline (PBS) with a pH of 7.4. This redox couple facilitated the monitoring of electron transfer processes at the electrode surface.

The electrophoresis buffer (1× TBE) was prepared with 0.1 M Tris, 0.1 M boric acid, and 2 mM EDTA, with a pH of 8.3. This buffer was used to maintain the proper ionic environment during gel electrophoresis, ensuring the effective separation of nucleic acids.

### 2.4. The Tox Gene Fragment Amplification in Standard PCR Mode

The standard PCR reaction for DNA amplification was performed using the Mastercycler AG 22331 thermal cycler with a total reaction mixture volume of 20 µL. The mixture consisted of 10 µL Hot Start Taq Master Mix containing Hot Start Taq polymerase, dNTPs, MgCl_2_, and KCl; 5 µL of primers at a concentration of 10 mM each; 2.5 µL template DNA isolated from a toxigenic strain of *Corynebacterium diphtheriae*; and 6.5 µL sterile, enzyme-free deionized water.

The PCR began with an initial step at 95 °C for 15 min. This was followed by 35 cycles of denaturation at 94 °C for 45 s, annealing at 60 °C for 45 s, and elongation at 72 °C for 45 s. The final elongation step was prolonged to 5 min. These parameters were applied according to the user’s manual. The resulting PCR products were then separated using electrophoresis on a 2% agarose gel.

### 2.5. The Tox Gene Fragment Amplification in Asymmetric PCR Mode (aPCR)

The goal of the asymmetric PCR (aPCR) was to obtain single-stranded DNA (ssDNA) complementary to the sequence of the probe immobilized on the sensor electrode. To achieve ssDNA, an excess of one primer is used in the aPCR reaction. The reaction mixture in this case included 20 µL of Hot Start Taq Master Mix, 0.25 µL of the forward primer at a concentration of 10 mM, 1.0 µL of the reverse primer at a concentration of 10 mM, 4.0 µL of template DNA, and 14.75 µL of sterile, enzyme-free deionized water.

## 3. Results and Discussion

### 3.1. PCR Microchip

#### 3.1.1. Design and Fabrication of PCR Microfluidic Device

**Figure 1:**
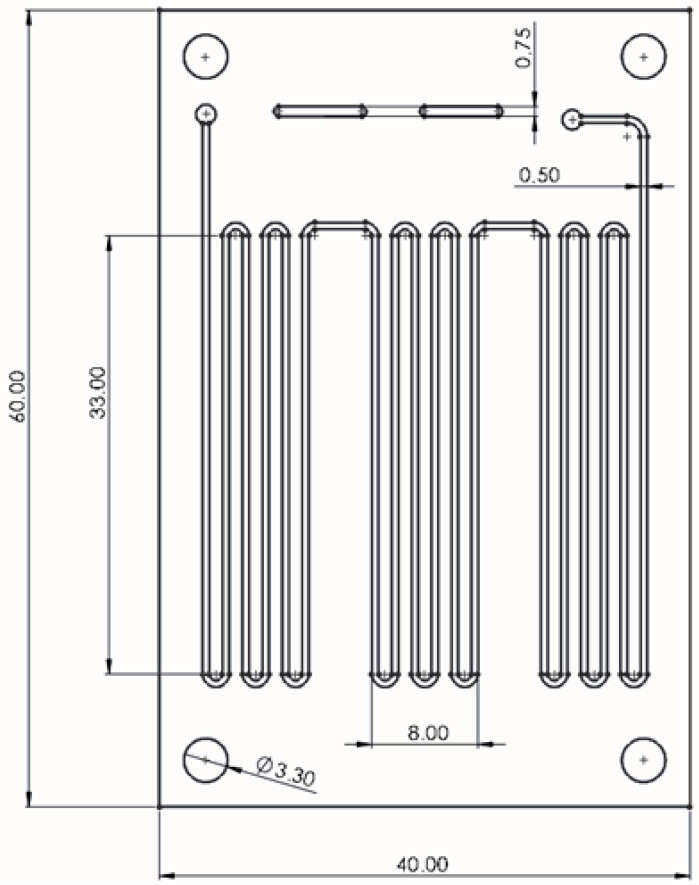
Design and dimensions of PCR microfluidic device.

The PCR microfluidic device consists of two main elements: a microreactor and a continuous microflow generating system. The microreactor is a serpentine microchannel divided into three zones in which the subsequent stages of the PCR reaction take place. The microchannel dimensions are 500 µm (W) × 100 µm (H) and the distance between each section of the channel in one zone is 1 mm. The total length of the microchannel is 700 mm. The distance between each temperature zone of the microreactor is 4.5 mm. The dimensions of the microchannel were experimentally determined in such a way as to ensure the residence time of the reactants in each zones at a constant flow value consistent with the values determined in the stationary reaction. The space between the individual zones of the microreactor was also experimentally determined using a thermal imaging camera. Figure 1 shows the microchannel design and its dimensions.

**Figure 2:**
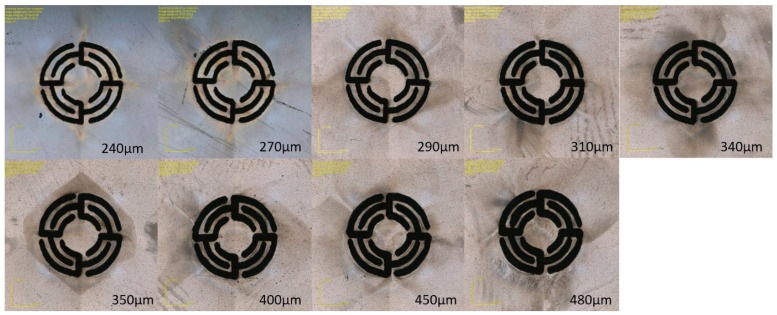
Planar springs with different dimensions.

Continuous flow is generated in the microchip by three check valves [12] and two pistons working alternately. The pistons, depending on the direction in which they move, generate underpressure and suck the solution from the microreactor or generate overpressure by pumping the solution into the microreactor. Check valves allows one to appropriately direct the flow of the solution. The key elements of the microvalve are a planar spring, a seal, a microchannel supplying liquid, and a microchamber. In the central point of the spring, there is a flat surface to which a Viton^®^ fluorine rubber gasket with a thickness of 150 µm and a diameter of 1.5 mm is glued. The purpose of the spring is to press the seal against the inlet. However, when a pressurized solution appears in the inlet channel, the planar spring bends and the valve opens. However, when the pressurized solution appears on the other side of the spring, the seal will be pressed against the inlet hole, thus blocking the flow in the opposite direction. Figure 2 shows planar springs of different sizes.

**Figure 3:**
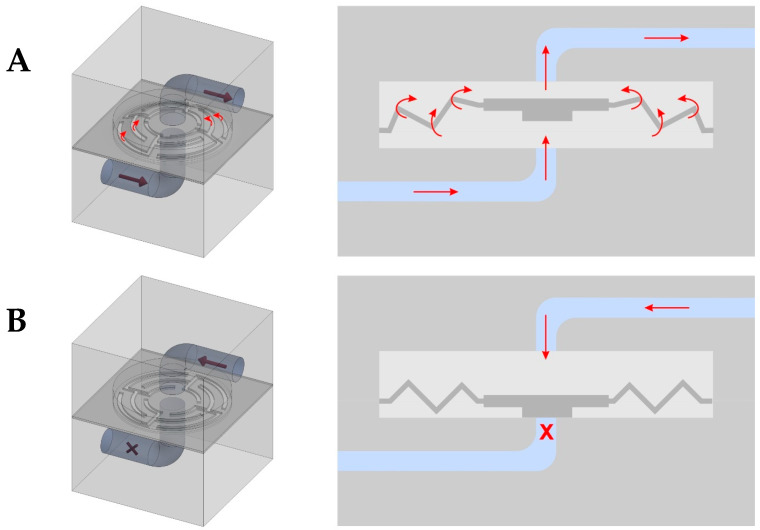
The operating principle of a planar spring valve. (**A**): The planar spring is pushed out by the fluid flow—the valve is open; (**B**): The planar spring is pushed back by the reverse flow towards the inlet—the valve is closed.

The size of the planar spring was experimentally optimized by measuring the pressure to the opening valves and measuring the back pressure, using a device with a pressure sensor. The operating principle of a planar spring valve is illustrated in Figure 3. Fluid flow in the intended direction forces the planar spring to expand, opening the valve (Figure 3A). In the event of reverse flow, the planar spring is pressed against the inlet port, closing the valve (Figure 3B).

**Figure 4:**
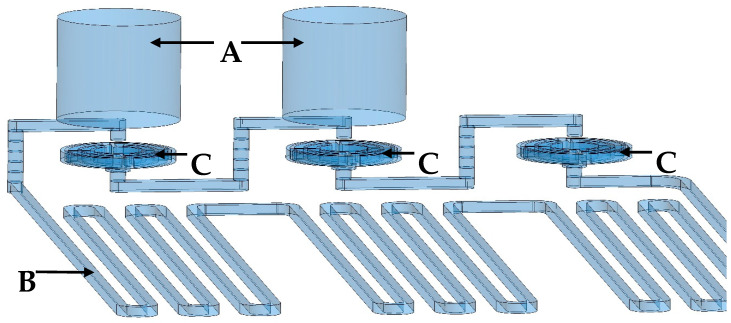
The main structures of the PCR microfluidic device. (**A**): Two structures milled in a top polycarbonate layer in which pistons move alternately, forcing the fluid flow; (**B**): The main microchannel in which the PCR reaction is carried out during the flow of the reaction mixture; (**C**): Planar springs acting as valves to prevent reverse flow.

PCR microfluidic device has a multi-layer structure. The main construction material of the chip is polyester foil in two variants: double sided adhesive polyester foil (3M9965) and double sided hydrophilic polyester foil (3M 9984). The layers of 3M 9984 and 3M9965 foils were made using laser-cutting techniques. For this purpose, a laser plotter with a CO_2_ laser with a maximum power of 30 W was used (Universal Laser Systems model VLS2.30 and Ultra X6000). Most often, this type of device uses a gas tube that generates a laser beam with a wavelength of 10.6 µm. Radiation at this wavelength is within the radiation absorption range of the polyester from which the foil layers are made. The use of such a wavelength of radiation leads to the melting of the polyester in the processing areas due to the absorption of radiation by the material. For this reason, laser radiation with a wavelength of 9.3 µm was used, which allowed to reduce radiation absorption and obtain smoother, melt-free edges in the cutting places. The microreactor is composed of three layers: the lower one is made of a double sided hydrophilic foil with is the bottom of the microchannels, the middle one is made of a double sided adhesive foil which contains the microchannels, and the upper one is made of a hydrophilic foil. The hydrophilic surface of microchannels has many advantages, the most visible of which is the easy and quick removal of air bubbles from the microchannels. In the next layers, microvalves were made, with planar springs made of a double sided hydrophilic foil layer. Additionally, there is a 5 mm thick polycarbonate layer on the top layer of the chip. This layer was made by milling on a CNC device (Datron Neo+). Design and milling program of the polycarbonate layer were made using SolidWorks and SolidCAM software. This layer serves several functions. It is an interface for connecting the pistons to the chip through holes manufactured in the layer, the diameter of which is identical to the diameter of the pistons. This layer also stiffens the chip structure, making it adhere precisely to the heating surfaces, making the energy exchange more efficient. Additionally, this layer also is an insulating layer that prevents the reaction zones from cooling down too quickly. Figure 4 presents the main structures of the PCR microfluidic device. Two pistons move alternately in two holes (Figure 4A) milled in the top layer causing a fluid flow through the main microchannel (Figure 4B) in which the PCR reaction occurs. Planar springs (Figure 4C) act as valves to prevent reverse flow in the system.

#### 3.1.2. Heating System and Temperature Control

**Figure 5:**
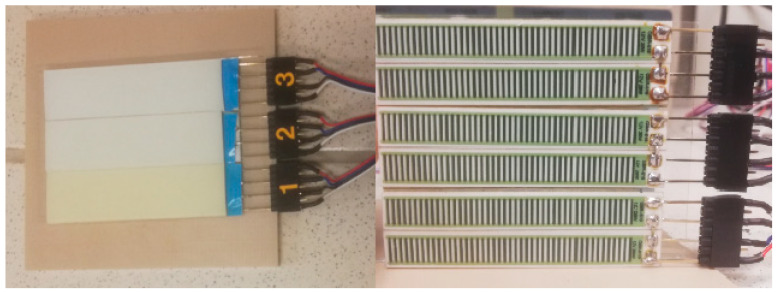
The heating system.

The heating system was composed of three pairs of ceramic heaters. Each pair was responsible for maintaining a set, constant temperature for a single zone corresponding to a given stage of the PCR reaction: a denaturation zone at 94 °C, a primer attachment zone at 55 °C, and elongation zone at 72 °C. The heating system used had the ability to control the temperature obtained by individual pairs of heaters thanks to platinum Pt 100 sensors (Swema, Farsta, Sweden). An aluminum plate was placed on the surface of each pair of heaters, which allowed for even temperature distribution over the entire surface of the pair of heaters. The heating system is shown in Figure 5.

**Figure 6:**
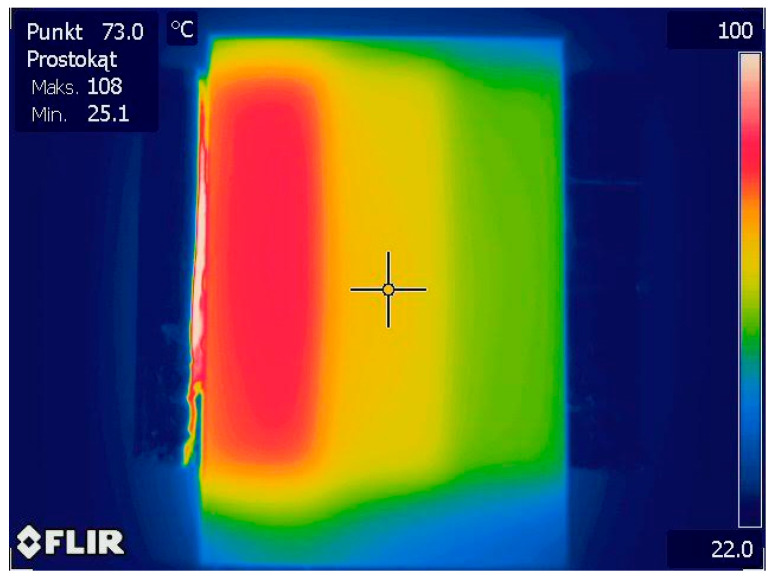
The temperature distribution on the heating surface.

The microfluidic devices were placed on the heaters in such a way that the individual temperature zones corresponded to the designed fragments of the microchannel geometry. Figure 6 shows the temperature distribution on the heating surface taken with a thermal imaging camera (Flir One^®^ Pro, Täby, Stockholm, Sweden).

Despite the chip design ensuring the best possible heat exchange between the heaters and the microchannels, the temperature of the reaction solution was lower than the temperature of the heating surfaces. The best solution to maintain a constant temperature of the reaction solutions would be to install temperature sensors inside the microchannels. However, such a solution would greatly complicate the construction of the chip. Therefore, it was decided to introduce appropriate corrections by comparing the temperature inside the microchannels with the temperature of the heating surfaces. For this purpose, a temperature measurement chip was constructed with microchannels in which Pt100 temperature sensors were placed. The results are presented in Table 1.

#### 3.1.3. Optimization of PCR Reaction

##### Flow Rate Optimization

The flow rate was optimized to ensure an adequate interaction time for each PCR stage while avoiding excessive residence times that could lead to non-specific amplifications or the degradation of reagents. In the microfluidic device, the flow rate was controlled by a combination of external syringe pumps and internal microvalves. The flow rates were optimized to balance the need for rapid thermal cycling with the requirement for sufficient reaction times within each thermal zone. This optimization was achieved through testing, adjusting the flow rates to maximize the yield and specificity of the PCR products. The solution flow rate in the microfluidic device was set to approximately 100 µL/min, which allowed for 30 reaction cycles to be carried out within 17.5 min. The total volume of the microfluidic device was calculated to be 59 µL (including 39 µL for the active microsystem and approximately 20 µL of dead volume).

##### Reaction Mixture Optimization

The concentration of PCR reagents, including primers, nucleotides, and polymerase enzyme were optimized for the use in microfluidic environment. Because of the small volumes and unique surface interactions within the microchannels, standard reagent concentrations used in conventional PCR may not be directly applicable. Reagent concentrations were varied to determine the optimal conditions that provided the highest amplification efficiency and specificity. Special attention was given to the enzyme’s performance at the microscale, ensuring that its activity remained consistent across the different thermal zones of the microfluidic device. Table 2 represents the concentration of the reaction mixture used to carry out reaction in the PCR microfluidic device.

#### 3.1.4. DNA and Polymerase Adsorption—Prevention Methods

The efficiency of PCR can be significantly affected by the adsorption of DNA and polymerase enzymes onto surfaces within the reaction environment. This phenomenon leads to a decrease in the concentration of these critical components, resulting in reduced amplification efficiency and the potential loss of target DNA sequences. Understanding and preventing DNA and polymerase adsorption are therefore crucial for optimizing PCR performance, particularly in microfluidic systems where the surface area-to-volume ratio is high. There are different ways that can prevent DNA and enzyme adsorption on a surface including surface coatings, surface passivation or usage of a blocking agents (which was used in this work) [13]. Bovine serum albumin (BSA) was used as a blocking agent. These agents work by occupying potential binding sites on the surfaces, thereby preventing the adsorption of the target molecules. BSA is particularly effective due to its high affinity for various surfaces and its ability to stabilize enzymes. Microfluidic devices shortly before their use for PCR reaction performance were filled with BSA solution and left for 1 h. After that, they were rinsed with nuclease free water.

#### 3.1.5. Electrophoretic Separation and Detection of PCR Products

Electrophoretic separation technique was used to help to detect the products of polymerase chain reaction (PCR), providing a means to verify the presence and size of amplified DNA fragments. This technique was used as an alternative for electrochemical detection of amplified ssDNA sequence. Figure 6 shows the product of PCR reaction, which was separated by electrophoresis.

#### 3.1.6. PCR Reaction Carried out in Thermocycler vs. in PCR Microfluidic Device

**Figure 7:**
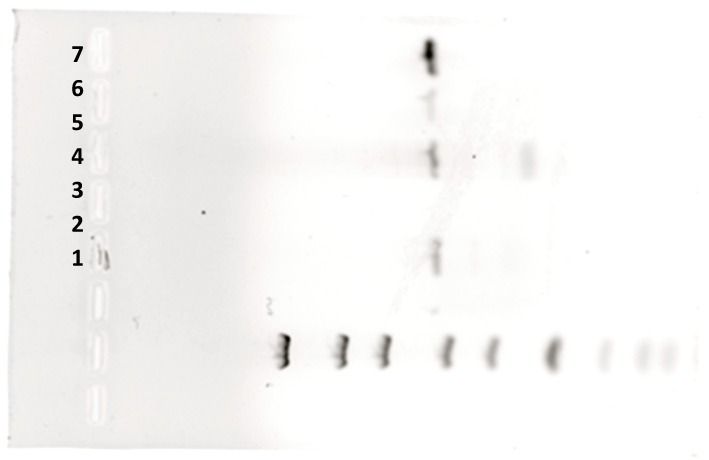
Photograph of an agarose gel after the electrophoresis process, showing DNA bands with specific strand lengths. 1—molecular ladder; 6—optimized reaction carried out in a PCR microfluidic device; 7—reaction carried out in a thermocycler (positive control); 2–5—other reactions carried out in various conditions.

Comparing PCR reaction carried out in a thermocycler vs. microfluidic device shows that thermal efficiency in a thermocycler relies on block heating and cooling process, which needs time. In contrast, a microfluidic device utilizes changes of temperature of the reaction mixture due to its flow through tree different temperature zones, ensuring rapid temperature changes. Reaction times are significantly shorter in microfluidic devices and were performed in less than 20 min due to efficient thermal management and reduced reaction volumes, compared to over 2 h which was required for a standard PCR protocol that was used when the reaction was carried out in a thermocycler. However, the efficiency of the reaction carried out in the thermocycler was higher than that of the reaction carried out in the PCR microfluidic device, which can be observed from the intensity of the bands in the agarose gel after the separation of the reaction products (Figure 7).

### 3.2. Electrochemical Sensor

In this paper, we developed a biosensor based on a “hairpin” molecular recognition mechanism. The biosensor relies on a single-stranded DNA (ssDNA) fragment modified at one end with a thiol group and at the other end with a redox marker, henceforth referred to as the probe. This probe solution was applied to the cleaned surface of a working electrode, where the thiol group formed a stable bond with the gold surface. A filler solution was subsequently added to enhance the organization of the receptor layer structure. The nucleotide sequence of the probe was designed to specifically bind to the target analyte. Each probe immobilized on the gold electrode contained a covalently attached electrochemical marker (methylene blue). In the absence of a complementary ssDNA fragment in the sample, the hairpin structure of the probe positions the redox marker near the electrode surface, resulting in detectable current responses. Upon hybridization between the ssDNA and its complementary sequence, the redox marker is distanced from the electrode, leading to a reduction in current intensity. The analytical signal was defined as the relative change in the electrochemical signal, calculated by the following equation:

(1) $$\mathrm{Signal}\;\mathrm{Change}=\frac{I_{0}-I}{I_{0}}\cdot 100\%$$

where *I*_0_ is the current intensity measured before hybridization, and I is the current intensity measured after hybridization.

The biosensor was fabricated in a similar manner as described in the paper “Molecular diagnostics of toxicogenic *Corynebacterium diphtheriae* strain by DNA sensor potentially suitable for electrochemical point-of-care diagnostics” [14]. The cited paper presents detailed information about the development of the biosensor and its analytical parameters and applications. The key distinction in the biosensor design lies in the type of transducer employed. In this study, a device fabricated on a flexible substrate was utilized, as detailed below.

#### 3.2.1. Fabrication of Biosensor’s Transducers on a Flexible Substrate

The biosensors were constructed using a three-electrode miniaturized transducer integrated with a multilayer PCR microfluidic device. The transducers, developed and supplied by the Printed Electronics, Textronics, and Assembly Department of the Center for Advanced Materials and Technology (CEZAMAT) at Warsaw University of Technology, were the three-electrode electrochemical transducers fabricated on flexible polymer film substrates. Conductive contacts, auxiliary electrodes, and reference electrodes were created using screen-printing technology, whereas the gold working electrodes were produced through thin-film deposition via physical vapor deposition.

#### 3.2.2. Biosensor Receptor Layer Preparation

The preparation of the biosensor began with the cleaning of the surface of the working electrode. The procedure was different than preparing the surface of the gold disk electrodes (often used in biosensor development [15]) due to the transducer’s small dimensions and even more importantly because they were developed as a disposable part of a system. Therefore, time-consuming steps such as polishing with alumina powder and ultrasonic treatment in demineralized water could be skipped. Ultimately, the cleaning procedure using piranha solution was selected as the most effective, after which the electrodes were abundantly rinsed with deionized water.

For probe immobilization on the electrode surfaces, an immobilization buffer containing 0.2 µM of a specific probe was applied for 30 s, after which the electrodes were rinsed with the same buffer. The electrodes were then incubated in immobilization buffer with 2 mM DEDTC/MCH for 24 h. Following this, they were rinsed with the immobilization solution and demineralized water, then placed in a hybridization buffer for 10 min.

#### 3.2.3. Rapid Detection of Tox Gene Fragment as a Product of Real Asymmetric PCR

**Figure 8:**
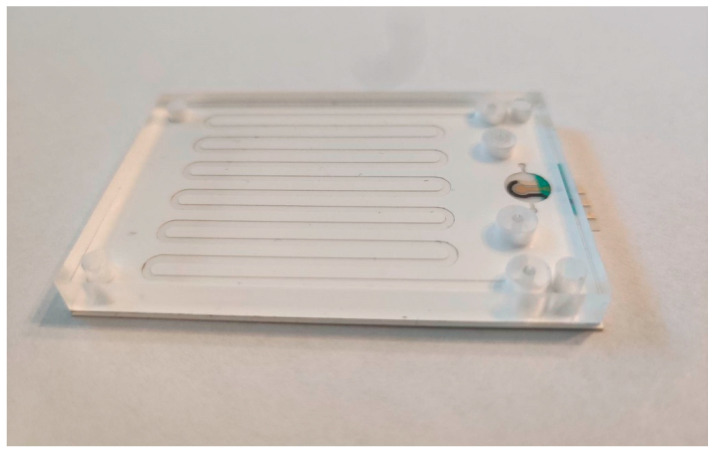
PCR microfluidic device integrated with a miniaturized DNA biosensor.

The developed biosensor was utilized to perform a ssDNA detection in the post-reaction mixture from the PCR microfluidic device. The microfluidic device was integrated with a miniaturized electrochemical biosensor (Figure 8). Initially, PCR was carried out in the microfluidic device, and then the post-reaction mixture was withdrawn and modified by the additional components of the detection buffer. The prepared reaction buffer was subsequently introduced into the cell containing the miniaturized biosensor, where the detection process occurred. The detection process in this experiment was performed this way because the flow system of the post-reaction mixture and its modification stage required additional, time-consuming research on process automation.

Two types of biosensors, differing in the blocking agent, were used in this experiment. One type of biosensor had MCH as the blocking agent, while the other had DEDTC. For the biosensor with MCH as the blocking agent, the post-reaction mixture was diluted twice with simultaneous addition of magnesium ions and DMSO to achieve a final concentration of 30% by volume according to the described procedure. The prepared mixture was then left in the biosensor cell for 30 min for hybridization to occur between the strands in the analyte and the receptor layer.

**Figure 9:**
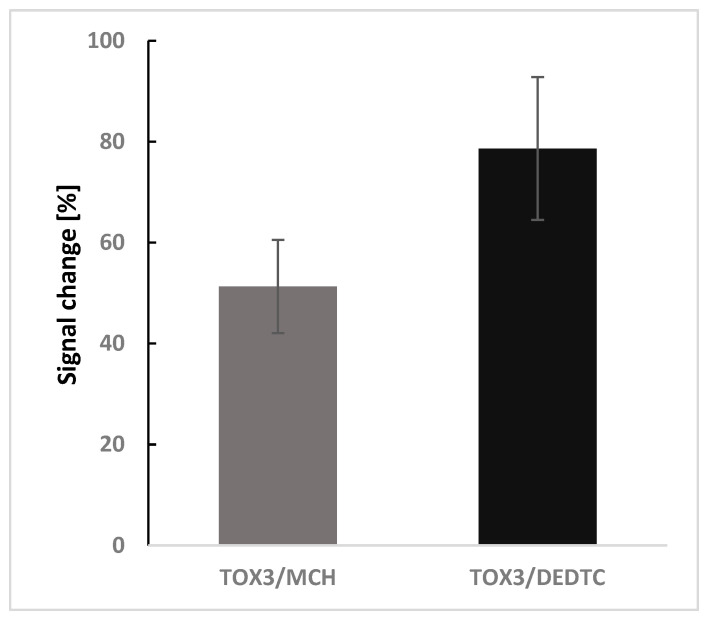
Signal change recorded by a biosensor containing different blocking agents in a receptor layer (MCH and DEDTC) fabricated on a miniaturized electrochemical transducer. The achieved signal was a result of an analytical detection of cDNA present in a post-reaction mixture (PCR carried out in the developed multilayer PCR microfluidic device); SWV, hybridization 30 min, n = 6.

A decrease in the registered current intensity was observed at 51.29% ± 9.78 (n = 6) for the TOX3/DEDTC biosensor and 78.64% ± 13.17 (n = 6) for the TOX3/MCH biosensor (Figure 9). The change in signal recorded by the biosensor before and after the hybridization process in the aPCR-containing buffer indicates the detection of cDNA in the tested sample. The differences between the two types of biosensors used may stem from several reasons. In the case of the TOX3/MCH biosensor, the reaction mixture is diluted, resulting in a lower cDNA concentration in the tested sample.

This confirms the ability of the developed device to detect ssDNA, which was amplified in the developed microfluidic using the developed biosensors. Despite the differences in the response of biosensors containing different blocking agents and differences in the buffer modification process, both variants enabled the detection of ssDNA present in the sample. In this paper, the purpose of the biosensor was to give a simple response that confirms or denies the presence of a diphtheria toxigenic species gene fragment in the sample without the need to specify its concentration. This basic functionality of the biosensor as a detection system has been confirmed.

## 4. Conclusions

In this paper, we showed the development of the PCR microfluidic device and electrochemical DNA biosensors using flexible foils and substrates for their fabrication. The paper demonstrates the potential of this PCR microfluidic device and biosensor system to deliver fast, accurate diagnostics in a portable, cost-effective form, laying groundwork for future POC diagnostic applications for other pathogens. Most importantly, the designed PCR microfluidic device enabled the rapid application of a toxigenic gen fragment of *Corynebacterium diphtheria*, and the biosensor integrated with it enabled its detection. The system constructed in this way allowed for the rapid detection of the pathogen, which may be an alternative to standard, time-consuming analytical procedures. The designed system was based on flexible strictures, which allowed for the integration of the PCR microfluidic device with the biosensor. Moreover, the flexible elements can be exploited for the production of such devices using, for example, roll-to-roll technology, which gives the potential for mass production of such systems.

## Figures and Tables

**Table 1:** Comparison of the temperature on the heating surface with the temperature inside the microchannels.

Temperature of the Heating Surfaces [°C]	Measurement of Temperature by Pt100 Sensors [°C]
101	97
75	73
56	55

**Table 2:** Composition of reaction mixtures used for conducting PCR reactions in the microfluidic device.

Reaction Mixture	Concentration
Primer F (5′-CAA TCA TCG TCA TAA TTT CCT TGT GTA CC-3′)	1250 nM
Primer R (5′-GAA AAC TTT TCT TCG TAC CAC GGG ACT AA-3′)	5000 nM
HotStartTaq Polymerase	X *
dNTP	200 µM
MgCl_2_	1.5 mM
Template DNA	≈0.023 ng/µL

* the manufacturer assures the optimal concentration of the polymerase for most reactions, without providing a specific value.

## Data Availability

All data will be available on request.
